# Synergistic Effect Induced by Gold Nanoparticles with Polyphenols Shell during Thermal Therapy: Macrophage Inflammatory Response and Cancer Cell Death Assessment

**DOI:** 10.3390/cancers13143610

**Published:** 2021-07-19

**Authors:** Valeria De Matteis, Mariafrancesca Cascione, Loris Rizzello, Daniela Erminia Manno, Claudia Di Guglielmo, Rosaria Rinaldi

**Affiliations:** 1 Department of Mathematics and Physics “Ennio De Giorgi”, University of Salento, Via Arnesano, 73100 Lecce, Italy; mariafrancesca.cascione@unisalento.it (M.C.); daniela.manno@unisalento.it (D.E.M.); ross.rinaldi@unisalento.it (R.R.); 2 Department of Pharmaceutical Sciences (DISFARM), University of Milan, Via G. Balzaretti 9, 20133 Milan, Italy; loris.rizzello@unimi.it; 3 National Institute of Molecular Genetics (INGM), Via F. Sforza 35, 20122 Milan, Italy; 4 Institute for Bioengineering of Catalonia (IBEC), The Barcelona Institute of Science and Technology, Baldiri Reixac 10-12, 08028 Barcelona, Spain; cdiguglielmo@ibecbarcelona.eu

**Keywords:** AuNPs, green synthesis, physico-chemical properties, polyphenols, thermal treatment, cancer, inflammation response

## Abstract

**Simple Summary:**

Polyphenols are present in a broad variety of plants, and they are known to possess anti-inflammation and anticancer properties. We used extracts from *Laurus nobilis* to synthetize gold nanoparticles (Au NPs), on which polyphenols were absorbed to form a stable shell (Au NPs@polyphenols). Then, the macrophage inflammation response was assessed, with the aim of using the Au NPs@polyphenols as synergistic tools in the thermal treatment of cancer cell models. We showed that the thermal conductivity enhancement, induced by Au during thermal treatment, increased the anticancer effects of polyphenols. After demonstrating the effectiveness of this system in in vitro cancer cell models, the future challenge will be the in vivo application of Au NPs@polyphenols.

**Abstract:**

Background: In recent decades, gold nanoparticle (Au NP)-based cancer therapy has been heavily debated. The physico-chemical properties of AuNPs can be exploited in photothermal therapy, making them a powerful tool for selectively killing cancer cells. However, the synthetic side products and capping agents often induce a strong activation of the inflammatory pathways of macrophages, thus limiting their further applications in vivo. Methods: Here, we described a green method to obtain stable polyphenol-capped AuNPs (Au NPs@polyphenols), as polyphenols are known for their anti-inflammatory and anticancer properties. These NPs were used in human macrophages to test key inflammation-related markers, such as NF-κB, TNF-α, and interleukins-6 and 8. The results were compared with similar NPs obtained by a traditional chemical route (without the polyphenol coating), proving the potential of Au NPs@polyphenols to strongly promote the shutdown of inflammation. This was useful in developing them for use as heat-synergized tools in the thermal treatment of two types of cancer cells, namely, breast cancer (MCF-7) and neuroblastoma (SH-SY5Y) cells. The cell viability, calcium release, oxidative stress, HSP-70 expression, mitochondrial, and DNA damage, as well as cytoskeleton alteration, were evaluated. Results: Our results clearly demonstrate that the combined strategy markedly exerts anticancer effects against the tested cancer cell, while neither of the single treatments (only heat or only NPs) induced significant changes. Conclusions: Au NP@polyphenols may be powerful agents in cancer treatment.

## 1. Introduction

Gold nanoparticles (Au NPs) have been employed in a wide range of applications due to their highly tunable physico-chemical features [1]. Surface plasmon resonance (SPR)—the oscillation of free electrons on the Au NP surface upon infrared radiation [2,3,4]—is definitely the hallmark of all Au NP optical properties. The heat conversion occurs when the plasmonic oscillation decays in a non-radiative manner. This behavior has been exploited, for example, in photothermal therapy (PTT), where malignant tissues are firstly loaded with Au NPs and then irradiated with infrared light [4]. The heat produced was able to significantly damage cancer cells and to induce tumor shrinking [5]. The best PPT-performing NPs are the Au-nanoshell and nanorods, as they are able to absorb the infrared light that deeply penetrate tissues [6]. Conversely, very small Au NPs (10 to 20 nm) cannot be used in PTT because of their absorption peak in the visible region [7]. Nevertheless, they can yield a thermal conductivity enhancement, reaching values of up to 23.8% and 21% at a concentration of 0.00026 vol% [8], after heat application. On the other hand, the enhancement of thermal conductivity by 4 nm Au NPs was c.a. 1.3%, using a concentration of 0.35 vol% in ethanol [9]. Thermal conductively can indeed be used for cancer treatment, without the use of radiation, also taking advantage of the high cell uptake rate of small NPs [10,11,12]. The broad biomedical applications of Au NPs can be ascribed to their high biocompatibility [13,14], together with the ease of surface modification, which is a crucial aspect for precise targeting [15,16].

Beyond the PTT, thermotherapy is also widely used in cancer treatment [17]. This methodology induces thermal stress using a temperature range between 41 and 45 °C in tumors, without exerting toxicity in healthy cells [18]. Thermotherapy is associated with chemotherapeutic drugs, with the aim of extirpating potential tumor cells that survive to the high temperature stress [19,20]. However, the drugs routinely used are rather toxic [21]. A good alternative is the application of natural anticancer compounds, like polyphenols [22]. Phenolic hydroxyl group-containing molecules indeed showed several anticancer effects, thanks to their antioxidant [23,24], anti-inflammatory [25], and anti-angiogenic properties [26,27]. They are commonly present in most plants, where three classes of polyphenols are the most abundant, namely, flavonoids, phenolic acids, and stilbenoids [28,29], all of which show a great ability to suppress tumor cell growth [30,31]. 

Flavonoids are characterized by a 15-carbon phenylpropanoid chain, forming two aromatic rings bounded by a heterocyclic pyran ring [32]. They modulate the reactive oxygen species (ROS)-scavenging enzymes, inducing cell cycle alteration due to the activation of caspases and finally inhibiting cancer progression and metastatization [33]. Phenolic acids are derivatives of benzoic and cinnamic acids and, as flavonoids, are potent antioxidants that are able to suppress tumorigenesis, acting as radical scavengers in the first phases of cancer progression [34]. 

Stilbenoids are constituted by a backbone stilbene structure, with different positions and chemical substituents on the ring [35]. They are efficient in the inhibition of cytochrome P450 (CYP) enzymes, preventing the first stage of tumorigenesis [36,37]. 

Furthermore, polyphenols behave as heat synergizes, improving the anticancer effect [38,39] by apoptosis stimulation and mitochondrial damage [40]. The synergistic use of these natural compounds and heat represents a new milestone in treating cancer tissues, while leaving healthy tissues untouched.

In addition, polyphenols have recently been used as reduction/capping agents to form metal-polyphenol networks by the coordination between Au ions and polyphenols [41], thus overcoming the conventional chemical routes, which require organic solutions containing metal precursors and toxic solvents [42]. The “green” routes for Au NPs synthesis are based on the use of polyphenols contained in plant extracts, allowing for the production of large-scale Au NPs [43]: the high yield, as well as the absence of toxic solvents, is particularly attractive for biomedical applications [44]. 

Among the plethora of plant species, *Laurus nobilis* is a native plant from the Mediterranean area, with well-known anti-inflammatory and antioxidant properties due to the high concentration of phenolic compounds (comprising the three classes mentioned above) [45,46], with an average concentration of 22 mg/L [47]. Compared with other plants, *Laurus nobilis* has a high antioxidant potential due to the high yield of polyphenols, which is c.a. 35% [48]. All the polyphenols contained in the *Laurus nobilis* extracts take part in the synthesis of metallic NPs. 

In this work, we used *Laurus nobilis* extracts to synthesize Au NPs. We achieved spherical NPs surrounded by a polyphenol shell (Au NPs@polyphenols). The polyphenols acted as reducing agents of the Au precursor, and at the same time, they covered the surface of the newly synthesized NPs, building up a nanometer-sized shell. We compared the green synthesis with a conventional approach, commonly used to obtain stable Au NPs, in order to understand the best material for thermal cancer treatment. 

Firstly, we explored whether incubation with Au NPs could trigger an inflammation response on macrophages by analyzing the nuclear translocation of the nuclear factor-κB (NF-κB), which is a typical marker of inflammation. We then used them for the treatment of cancer cells (MCF-7 and SHSY-5Y) in combination with a heat therapy (43 °C). The cell uptake, viability, calcium release, oxidative stress, HSP-70 expression, mitochondrial swelling, DNA damage, and actin alterations induced by the combined treatment (thermal and Au NPs@polyphenols) were evaluated. We showed how the Au NPs-induced heating improves the anticancer effects of polyphenols absorbed on the surface of NPs. This effect was not detected in cells treated with either heat or polyphenols independently. We believe that these results could contribute to the development of next-generation combinatorial cancer therapy based on nanostructured materials.

## 2. Materials and Methods

### 2.1. Synthesis of AuNPs by Colloidal and Green Routes

#### 2.1.1. Colloidal Route

Colloidal syntheses of the Au NPs were performed according to the procedure described in [49], who showed a metal salt reduction in aqueous solution using sodium citrate (Sigma-Aldrich, Dorset, UK). A reaction flask, filled with 150 mL of 0.25 mM of HAuCl_4_ (Sigma-Aldrich, Dorset, UK) aqueous solution, was heated to the boiling point under reflux while stirring, and then sodium citrate was rapidly injected. The solution in the flask was kept at the boiling point, until the color solution became wine red. The reaction solution was then cooled down to room temperature and stored in the dark at 4 °C. After cooling, the solution was centrifuged at 7500 rpm for 45 min and washed three times with MilliQ water to remove the residuals. 

#### 2.1.2. Green Route

##### Preparation of Leaf Extracts

Leaves of *Laurus nobilis* were collected in winter. After several washes with MilliQ water to remove contaminants, the leaves were dried at room temperature for 24 h. Then, 25 g of leaves were finely cut and added to a beaker containing 250 mL of MilliQ water. The mix was boiled at 100 °C for 20 min, and it was successively cooled down to room temperature. The extract was filtered with Whatman No. 1 filter paper before use for NPs synthesis. For the incubation in cells, the solution was sterilized using a freeze-drying method.

##### Green Synthesis of AuNPs 

First, 5 mL of purified extract was added to a solution containing HAuCl_4_ dissolved in MilliQ water (1 mM) to obtain a 1:4 ratio between the extract and HAuCl_4_ water solution. The mixture was subjected to stirring (300 rpm) for 1 h at room temperature, until the color of the reaction solution turned from light brown to wine red. The solution was centrifuged for 1 h at 4000 to collect the NPs in the pellet, which was purified with MilliQ water by three cycles of centrifugation. Finally, the NPs were concentrated by an Amicon Ultra Centrifugal 3k Filter (Sigma Aldrich, Dorset, UK).

### 2.2. Total Phenolic Compound Content Estimation

The Folin-Ciocalteu phenol reagent was used to measure the total phenolic content in the leaf extract and in a solution containing the Au NPs obtained by a green route, following the method previously described by Singleton and Rossi, with some modifications [50]. Briefly, 1.8 mL of the Folin-Ciocalteu reagent and sodium bicarbonate (7.5%) was successively added to 40 μL of leaf extract at room temperature. The mixture was kept in the dark for 1 h, and then the absorption spectra were recorded at 765 nm using a Jasco V-630 spectrophotometer. Gallic acid was used as the standard phenolic compound for the calibration curve (r^2^ = 0.9989). The total phenolic compound content is expressed as the residual gallic acid equivalents (mg/L of the extract). The data are expressed as the mean ± SD. 

### 2.3. Characterization of AuNPs

Transmission electron microscopy (TEM) images were obtained using a HITACHI 7700 transmission electron microscope operating at 120 Kv. The solutions containing NPs were dropped onto a standard carbon supported 600-mesh copper grid. The DLS and ζ-potential acquisitions were recorded by a Zetasizer Nano-ZS, with a HeNe laser (4.0 mW) working at 633 nm detector (ZEN3600, Malvern Instruments Ltd., Malvern, UK), in aqueous solutions (25 °C, pH 7). The NP size statistical distribution was measured on 100 Au NPs and green Au NPs@polyphenols fitted by a normal Gaussian function. The surface plasmon responses of different AuNPs were recorded using a Shimadzu-2550 with 1 cm quartz cuvettes. The FTIR spectra over a range of 400–4000. cm^−1^ of the pelletized samples were obtained at a resolution of 4 cm^−1^ using a Jasco-670 Plus FTIR spectrometer (Jasco, Tokyo, Japan). 

### 2.4. THP-1 Culture and Differentiation

Human leukemic monocytes (THP-1) (ATCC) were cultured and maintained in RPMI-1640, containing 2 mM l-glutamine and 25 mM Hepes (Sigma-Aldrich, Dorset, UK), and supplemented with 10% (*v/v*) heat-inactivated fetal bovine serum (FBS, Sigma-Aldrich, Dorset, UK), 1% (*v/v*) penicillin-streptomycin (Sigma-Aldrich, Dorset, UK), and 0.1% (*v/v*) amphotericin B (Sigma-Aldrich, Dorset, UK). The THP-1 cells were used for in vitro experiments of passage numbers nine and twenty. The in vitro experiments with these cells were carried out between the passage numbers three and nine. Prior to the in vitro cellular studies, THP-1 cell differentiation into a mature macrophage-like state (M0-macrophages) was induced through incubation with 10 ng/mL of phorbol 12-myristate 13-acetate (PMA, Sigma-Aldrich, Dorset, UK) for 48 h in a humidified atmosphere, with 95% air and 5% CO_2_, at 37 °C.

### 2.5. MCF-7 and SH-SY5Y Culture

Human breast cancer cells (MCF-7, ATCC^®^ HTB-22™) and human neuroblastoma cells (SH-SY5Y, ATCC^®^ CRL-2266™) were maintained in Dulbecco’s Modified Eagle Medium (DMEM) (Sigma-Aldrich, Dorset, UK) with 50 μM of glutamine, supplemented with 100 U/mL of penicillin/streptomycin (Sigma-Aldrich, Dorset, UK) and 100 mg/mL of 10% Fetal Bovine Serum (FBS) (Sigma-Aldrich, Dorset, UK). The cells were incubated in a humidified controlled atmosphere with a 95 to 5% ratio of air/CO_2_ at 37 °C.

### 2.6. Cell Viability Assay on THP-1, MCF-7 and SH-SY5Y at 37 °C

The THP-1 were seeded at a concentration of 5 × 10^3^ cells per well in 96-well plates (CytoOne) and differentiated as mentioned above (Section 2.3). 

First, 1 μM (c1) and 3 μM (c2) concentrations of Au NPs and Au NPs@polyphenols were then incubated for 24 and 48 h in a humidified atmosphere, with 95% air and 5% CO_2_, at 37 °C. Control wells were incubated with equivalent volumes of a cell culture medium and/or solution of 10% (*v/v*) dimethyl sulfoxide (DMSO, Sigma-Aldrich, Dorset, UK) in DPBS. MCF-7 and SH-SY5Y were seeded in 96-well microplates. After 24 h of stabilization, the cells were exposed to c1 and c2 of Au NPs@polyphenols for 24 h and 48 h. The same procedure was used to quantify the viability of using polyphenol extract and AuNPs at c1 and c2 concentrations for 24 h and 48 h. 

A WST-8 (Sigma-Aldrich, Dorset, UK) assay was performed following the procedure previously described in [51]. The data were expressed as the mean ± SD. 

### 2.7. Inductively Coupled Plasma Emission Spectroscopy (ICP-OES) 

#### 2.7.1. NPs Concentration

The concentrations of the Au NPs and Au NPs@polyphenols were calculated by elemental analyses using an ICP-OES Perkin Elmer AVIO 500. A total of 250 μL of the Au NP and Au NPs@polyphenol solutions were digested overnight by adding 2 mL of *aqua regia* (HCL:HNO_3_, 3:1), followed by dilution with MilliQ water (1:5).

#### 2.7.2. Cell Uptake

First, 1 × 10^5^ of THP-1 cells (after differentiation) were seeded in 1 mL of the medium in a 6-well plate. After 24 h at 37 °C, the medium was replaced with a fresh medium containing the obtained Au NPs and Au NPs@polyphenols at a concentration of 1 μM (c1) and 3 μM (c2). After 24 h and 48 h of incubation at 37 °C, the culture medium was removed, and the cells were washed with PBS to eliminate non-internalized NPs. The cells were detached with trypsin and counted using an automatic cell counting chamber. Then, 360,000 cells were suspended in 200 μL of MilliQ water and digested by *aqua regia*. After dilution with MilliQ water, the solution was analyzed to evaluate the Au content. Elemental analysis was carried out using an ICP-OES Perkin Elmer AVIO 500. The same procedure was conducted to evaluate the uptake of Au NPs@polyphenols in SH-SY5Y and MCF-7 cell lines at c1 and c2 concentrations for 24 h and 48 h. 

### 2.8. Cytokines Responses

The release of cytokines IL-6 and IL-8 and the growth factor, TNF-α, was quantified by an enzyme-linked immunosorbent assay (ELISA) on THP-1 (after differentiation) exposed to 1 μM (c1) and 3 μM (c2) of Au NPs and Au NPs@polyphenols for 24 h and 48 h. 

After the centrifugation step (2000× *g* for 10 min), the supernatants from the cultures containing 0.5 × 10^6^ cells/mL in a final volume of 1 mL were collected and stored at −80 °C until the analyses. Human IL-8 and IL-6 and TNF- α ELISA kits (Abcam, Cambridge, UK) were used, following the manufacturing procedure, after the calibration curve construction. The reactions were quantified by spectrophotometry. 

### 2.9. NF-κB Signaling Imaging and Quantification Assay

NF-κB signalling imaging was preformed using a Confocal Laser Scanning Microscope (CLSM, Leica SP8, Milton Keynes, UK). Firstly, the THP-1 cells were seeded at a concentration of 5 × 10^4^ cells per glass-bottom Petri dish (Ibidi) and differentiated as mentioned above. Then, the M0-macrophages were incubated with 1 μM (c1) and 3 μM (c2) of Au NPs and Au NPs@polyphenols for 24 h and 48 h in a humidified atmosphere, with 95% air and 5% CO_2_, at 37 °C. Following the treatment, the cells were washed with DPBS and fixed using 3.7% formaldehyde (Sigma-Aldrich, Dorset, UK) for 10 min at room temperature (RT). After the fixation step, followed by DPBS washing for the membrane permeabilization step, the cells were incubated with 0.2% Triton-X (Sigma-Aldrich, Dorset, UK) for a further 10 min at RT. Then, immunostaining blocking was performed, using 5% bovine serum albumin (BSA) (Sigma-Aldrich, Dorset, UK) to prevent unspecific antibody binding. After 1 h at RT, the cells were incubated with NF-κB p65 Antibody (F-6) and FITC (Santa Cruz Biotechnology Inc., Heidelberg, Germany) diluted in 1% BSA overnight in a humidified chamber at 4 °C. After 24 h, the cells were washed with DPBS, and the nuclei were stained with Hoescht 33342 (Sigma-Aldrich, Dorset, UK) for 10 min at RT. At least 10 different regions of the petri dishes were acquired by a confocal microscope, and an NF-κB nuclear translocation imaging analysis was evaluated by co-localisation (Pierce’s coefficient values) of the NF-κB and nucleus fluorescence intensity signals using the Fiji ImageJ software (version 2.0). The plasma membrane was stained using a CellMask™ Deep Red Plasma membrane Stain (Thermo Fisher Scientific, Waltham, MA, USA), and nuclei were stained with Hoechst 33342 (Thermo Fisher Scientific, Waltham, MA, USA). 

### 2.10. Thermal Treatment

Hyperthermal stress of MCF-7 and SH-SY5Y was induced by increasing the incubator temperature to 43 °C, as previously reported in [52], with and without the treatment with 1 μM (c1) and 3 μM (c2) of Au NPs@polyphenols for 24 h and 48 h. After 45 min of exposure, the cells were reported at a temperature of 37 °C for 12 h, before the further assays and confocal imaging. In this way, the assays reported, and the confocal images were conducted in heat-treated cells and without heat exposure as a control. Another control was constituted by cells incubated with c1 and c2 of only polyphenols at temperatures of 37 °C and 43 °C for 24 h and 48 h, and the cells were only exposed to Au NPs at 37 °C and 43 °C for 24 h and 48 h.

### 2.11. Viability Assay on SHSY-5Y and MCF-7 at 43 °C

After the thermal treatment described in 2.10, the SH-SY5Y and MCF-7 cell viability was investigated using a standard WST-8 assay, following the procedure previously described. The negative controls are constituted by untreated cells exposed to 43 °C, and cells treated with 1 μM (c1) and 3 μM (c2) of polyphenols at 43 °C for 24 h and 48 h.

### 2.12. Calcium Assay

The MCF-7 and SH-SY5Y cells were seeded at a concentration of 7 × 10^4^ cells/mL in glass Petri dishes. After 24 h of stabilization, the culture media was removed and replaced with solutions of 1 μM (c1) and 3 μM (c2) of Au NPs@polyphenols for 24 h and 48 h. After this, some samples were used to induce thermal stress by the procedure previously described (ph. 2.8). Then, all the samples (physiological and thermal-induced) were homogenized by sonication in 100 μL of lysis buffer (100 mM Tris, pH 7.5) to quantify the intracellular Ca^2+^ concentration. The homogenates were centrifuged at 10,000× *g* for 15 min at 4 °C. The supernatant was collected and stored on ice, and the samples were analyzed on the same day using a Ca^2+^ Assay Kit (Abcam, ab102505, Cambridge, UK), following the manufacturer’s instructions. The calcium release amount is expressed relative to the untreated cells. In the calcium assay protocol, a chromogenic complex is formed between the calcium ions and 0-cresolphthalein. The complex is measured at OD = 575 nm

### 2.13. Oxidative Stress Assay

The SH-SY5Y and MCF-7 cells were seeded in 96-well microplates and treated with 1 μM (c1) and 3 μM (c2) of Au NPs@polyphenols for 24 h and 48 h. After these time points, some samples were used to induce thermal stress by the procedure previously described (ph. 2.8). Then, the 2’,’-Dichlorodihydrofluorescein diacetate (DCFH-DA) (Sigma-Aldrich, Dorset, UK) assay was performed on microplates, following the procedure reported in [32]. The data were expressed as the mean ± SD. Additionally, the green fluorescence of ROS was visualized using fluorescent microscopy.

### 2.14. Heat Shock Protein 70 (HSP-70) Assay

The SH-SY5Y and MCF-7 cells were seeded in 96-well microplates and treated with 1 μM (c1) and 3 μM (c2) of Au NPs@polyphenols for 24 h and 48 h. After these time points, some samples were used to induce thermal stress by the procedure previously described (ph. 2.8). Then, the cells were lysed, and the HSP-70 ELISA kit (Abcam, ab133060; Abcam, Cambridge, MA, USA) was used, following the manufacturer’s instructions, to build a calibration curve. The samples were read at 450 nm, which is proportional to the amount of analytes bound. 

### 2.15. Mitochondrial Membrane Potential and Mitochondrial Morphology Studies by Confocal Microscopy and Imagej Software 

The MCF-7 and SH-SY5Y cells were exposed for 48 h to 3 μM (c2) of Au NPs@polyphenols, and thermal treatment was applied, as reported in 2.10. After the treatment, the NPs were removed, and the cells were washed several times with PBS and, finally, incubated with 300 nM mL^−1^ of MitoTracker™ Deep Red (Thermo Fisher Scientific, Waltham, MA, USA) to label the mitochondria. After 20 min in the incubator, the confocal acquisitions were collected by the Leica TCS SPE-II confocal microscope, using a ×100 objective (water immersion, HCX PL APO, 1.10NA). The average mitochondrial circularity and mitochondria area/perimeter normalized to circularity were measured on 20 cells by means of the ImageJ 1.47 analysis software. 

### 2.16. Mitochondrial Membrane Potential Assay (JC1 Assay)

The SHSY-5Y and MCF-7 cells were seeded in black 96-well microplates and treated with 3 μM (c2) of Au NPs@polyphenols for 48 h. Then, the cells were exposed to thermal treatment, as reported in 2.10. Successively, the mitochondrial membrane potential assay was performed using JC-1 (Thermo Fisher Scientific, Waltham, MA, USA), a cationic carbocyanine dye that accumulates in mitochondria. The analysis was conducted on microplates, following the manufacturer’s instructions, using a Fluo Star Optima microplate reader (BMG LABTECH, Offenburg, Germany). After NPs exposure, the medium was removed, and the cells were firstly washed with PBS and then incubated with JC-1 (2.5 μg/mL) for 2 h. After removing the dye, the cells were washed, and the fluorescence of the cells from each well was measured and recorded. The results are normalized with respect to the negative controls (expressed as 100%). As a positive control, the cells were incubated with 100 μM of valinomycin.

### 2.17. Comet Assay (Single Gel Electrophoresis)

The MCF-7 and SH-SY5Y were incubated with 3 μM (c2) of Au NPs@polyphenols for 48 h at a density of 5 × 10^4^ in each well of 12-well plates in a volume of 1.5 mL. Then, the cells were exposed to thermal treatment, as reported in 2.10. After the treatments, the cells were centrifuged and suspended in 10 μL of PBS at concentration of 1000 cells/μL. The cell pellets were mixed with 75 μL of 0.75% low-melting-point agarose (LMA) and then layered on microscope slides pre-coated with 1% normal melting agarose (NMA) and dried at room temperature. Subsequently, the slides were immersed in an alkaline solution (300 mM of NaOH and 1 mM of Na_2_EDTA, pH 13) for 20 min to allow for the unwinding of the DNA. The electrophoresis was carried out in the same buffer for 25 min at 25 V and 300 mA (0.73 V/cm). After electrophoresis, the cellular DNA was neutralized by successive incubations in a neutralized solution (0.4 M Tris–HCl, pH 7.5) for 5 min at room temperature. The slides were stained with 80 μL of SYBR Green I (Invitrogen). The comets derived from single cells were photographed under a Nikon Eclipse Ti fluorescence microscope, and the head intensity/tail length of each comet was quantified using the Comet IV program (Perceptive Instruments). A positive control was obtained for incubated cells using H_2_O_2_ (100 μM). 

### 2.18. Confocal Analysis and Morphometric Quantifications of MCF-7 and SH-SY5Y

MCF-7 and SH-SY5Y cells were seeded at a concentration of 7 × 10^4^ cells/mL in glass Petri dishes. After 24 h of stabilization, the culture media was removed and replaced with Au NPs@polyphenols at 3 μM (c2) for 48 h. The cells were exposed to thermal treatment, as reported in 2.10. After this time, the NP solutions were removed, and the cells were washed with PBS. The samples were fixed using glutaraldehyde (0.25%) for 10 min, and then the cells were permeabilized by Triton X-100 (0.1%) for 5 min. F-actin was stained using 1 μg/mL of phalloidin-FITC overnight. Acquisitions were performed by a Zeiss LSM700 (Carl Zeiss Microscopy GmbH, Munich, Germany) CLSM mounted on an Axio Observer Z1 (Carl Zeiss Microscopy GmbH Munich Germany) inverted microscope, using the Alpha Plan-Apochromat (Carl Zeiss Microscopy GmbH, Munich Germany) 100× oil-immersion objective with 1.46 NA2.10. Morphometric quantification (coherency of F-actin) was performed on 15 cells using the OrientationJ plugin of the ImageJ 1.47 software. This parameter indicates the local orientation of actin filaments. In particular, the value of coherency ranges from 0 (isotropic orientation) to 1 (perfectly oriented structures) [53].

### 2.19. Statistical Analysis

Statistical analyses were performed using OriginPro (version 8.1). The difference between three and more groups was analyzed through one-way or two-way ANOVA multiple comparisons, respectively. The differences between two groups were evaluated by a two-tailed Student’s-test. The differences were statistically significant when * *p* < 0.05, ** *p* < 0.01, *** *p* < 0.001).

## 3. Results and Discussion 

**Figure 1:**
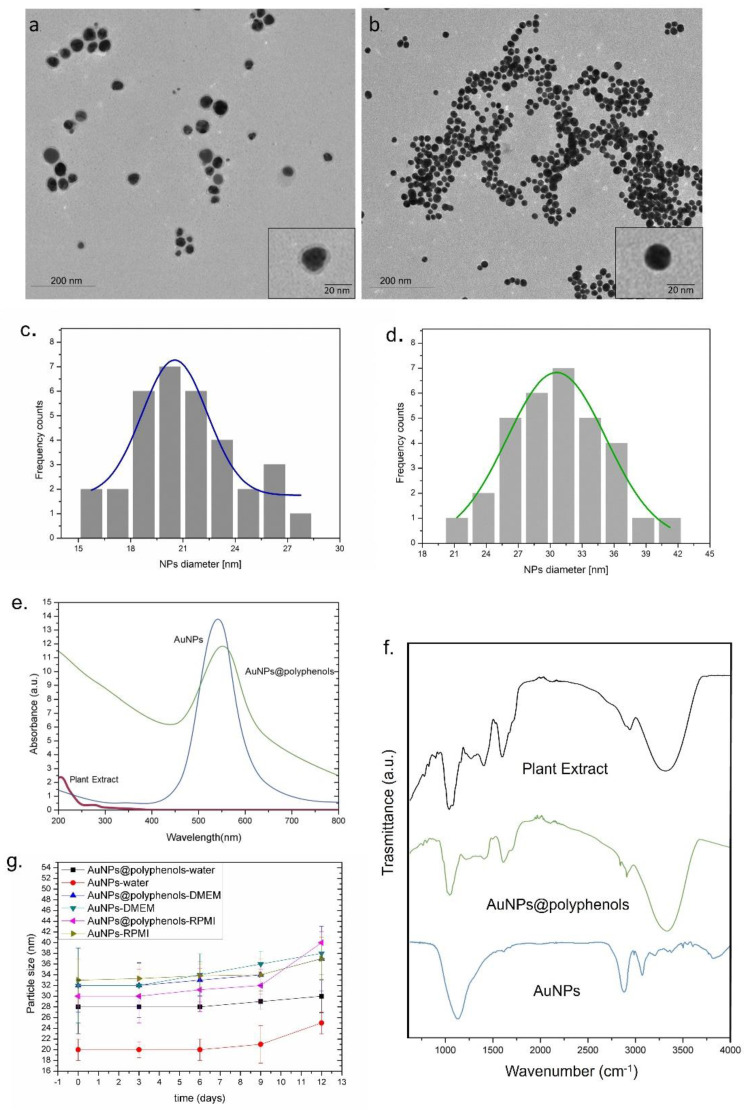
Representative TEM images of Au NPs@polyphenols (**a**) and conventional Au NPs (**b**); Statistical analysis with Gaussian fit (black line) for Au NPs@polyphenols (**c**) and Au NPs (**d**); UV-vis (**e**) and FTIR measurements (**f**) conducted on plant extract, Au NPs@polyphenols and Au NPs. Stability studies (**g**) of Au NPs@polyphenols and conventional Au NPs in water, DMEM and RPMI, measured by DLS and acquired after 0, 3, 6, 9, and 12 days. The concentration used was 2 μM.

Polyphenols are particularly fascinating for their strong antiapoptotic and anticancer effects [54,55,56,57,58,59,60,61]. Recently, it has been demonstrated that polyphenols can be used for successfully synthetizing metallic NPs [62], such as Au NPs, as they can act as reducing and capping agents. The metal-polyphenols network, formed by the interactions between polyphenols and metal ions, was useful to achieve a novel class of polyphenols containing NPs for drug delivery, imaging, and disease treatment. In this connection, there are different chemical pathways that can occur during the synthesis processes, such as: (i) a covalent interaction, including a Baeyer reaction, (ii) a catechol-boronate interaction, (iii) a metal chelation, (iii) a Schiff base reaction, and (iv) non-covalent interactions, including hydrogen bonding, hydrophobic, and electrostatic interactions [28]. In our work, we used *Laurus nobilis* plant extracts to obtain Au NPs, because these plants are widely spread in the Mediterranean area and represent a theoretically unlimited source of polyphenols. Similar NPs were obtained using a conventional colloidal route for comparison studies. The TEM images (Figure 1a) showed the presence of polyphenols absorbed on the Au NP (Au NP@polyphenol) surface as a clear visible low-contrast organic shell, which was absent in the Au NPs obtained by the conventional Turkevich–Frens method [63,64] (Figure 1b). These differently synthetized NPs were compared in terms of triggering different toxicological and immunological pathways. In terms of synthesis, both chemical methods allowed for obtaining spherical NPs with a mean size distribution of (28 ± 5) and (20 ± 2) nm for Au NPs@polyphenols or standard synthesis, respectively (by ImageJ-based analyses). The statistical analysis was conducted by selecting 70 nano-objects for each NP performing a Gaussian fitting (Figure 1c,d). The size measurements for Au NPs@polyphenols were conducted, excluding the shell of polyphenols, which have a mean size of about (7 ± 3) nm.

To understand the quantity of polyphenols involved in the green synthesis, the total polyphenol concentration was measured on plant extract and on the Au NP@polyphenol water solution by the Folin-Ciocalteu method. We quantified (27 ± 3.7) mg/L from the plant extract and (15 ± 2.4) mg/L from the Au NP@polyphenol samples. The TEM images and the total polyphenols measurements confirmed the presence of polyphenols on the Au NPs (Table 1).

DLS measurements on Au NPs@polyphenols (in water) confirmed the TEM data and showed NPs with a hydrodynamic radius of (28 ± 5) nm. Conversely, the Au NPs synthetized by the standard chemical route had a size of (20 ± 2) nm. The negative surface charge was observed for both types of NPs, namely, (−48 ± 3) mV and (−40 ± 4) mV for Au NPs@polyphenols and conventional Au NPs, respectively (Table 2). DLS and zeta potential characterizations were also performed in the cell culture growth media, RPMI, and DMEM. We observed an increase in the NPs size in the Au NPs, which was most probably due to the protein corona formation and is in line with previous evidence [65,66]. For Au NPs@polyphenols, the effect was negligible, and this was probably due to the polyphenol shell, which can induce steric hindrance and a more negative surface (Table 2), potentially reducing the binding with serum proteins. 

The UV-Vis spectra of Au NPs@polyphenols and conventional Au NPs a localized surface plasmon resonance (LSPR) absorption peaks at c.a 556 nm and 550 nm, respectively. The spectra related to both experimental groups did not overlap with the UV-vis measurements conducted on plant extracts (Figure 1e).

An FTIR measurement was carried out to study the potential NP interactions and to characterize the capping agent. Firstly, we examined the FTIR spectra of the extract and observed strong bands at 3353, 1633, and 1225 cm^−1^ corresponding to the O–H stretching vibration of alcohols and phenols, C=O stretching vibration of tertiary amides, and C–O stretching of aromatic ethers (Figure 1f). The IR spectra of Au NPs@polyphenols showed bands at 3270, 1651, and 1023 cm^−1^ corresponding to the O–H stretching vibration of alcohols and phenols, C=O stretching vibration of tertiary amides, and C–OH stretching of primary alcohols. The Au NPs exhibited bands at 3285, 1633, and 1025 cm^−1^ related to the O–H stretching vibration of alcohols and phenols, C=O stretching vibration of tertiary amides, and C–OH stretching of primary alcohols. This suggested that the *Laurus nobilis* polyphenols are responsible for the NP surface coordination and, consequently, are involved in their water stabilization. We observed a peak at 677 cm^−1^ for the Au NPs obtained by the conventional method corresponding to the M–O (M = metal; O = oxygen) stretching vibration, confirming the presence of citrate ions via RCOO–Au coordination. The presence of citrate was confirmed by the peaks corresponding to R–CO_2_ stretching and C–O stretching at 1540 and 1236 cm^−1^, respectively. The peaks of 3700 were ascribed to the stretching vibrations of O–H. We then assessed the stability of Au NPs@polyphenols and Au NPs after 3, 6, 9, and 12 days by DLS (Figure 1g). As expected, the size and particle distribution did not change up to the 6th day, when the aggregation was observed, which was probably due to the culture medium serum, salts, and vitamins. 

After characterizing the physico-chemical properties of the two NPs, we investigated their potentially different impact on human monocytes (THP1) and differentiated the primary macrophage (M0) phenotype by PMA incubation. Macrophages (M0) can be polarized toward pro-inflammatory (M1) or anti-inflammatory (M2) phenotypes, which play completely different roles in immune regulation and inflammation [67]. 

We started evaluating the viability of M0 macrophages exposed to 1 μM (c1) and 3 μM (c2) of Au NPs@polyphenols and conventional Au NPs for 24 and 48 h. The rationale behind these experiments was to assess whether NPs could impact cell viability or activate inflammation in vitro, both of which could limit further pre-clinical investigations.

The concentration of NPs was chosen based on our previous studies, in which a concentration greater than 3 μM was used, and the NPs were not much more stable in the cell culture medium undergoing aggregation phenomena. Concentrations below 1 μM were not considered for the studies, as a strong induction of macrophages was imperative to understand which of the two types of NPs induced inflammation.

**Figure 2:**
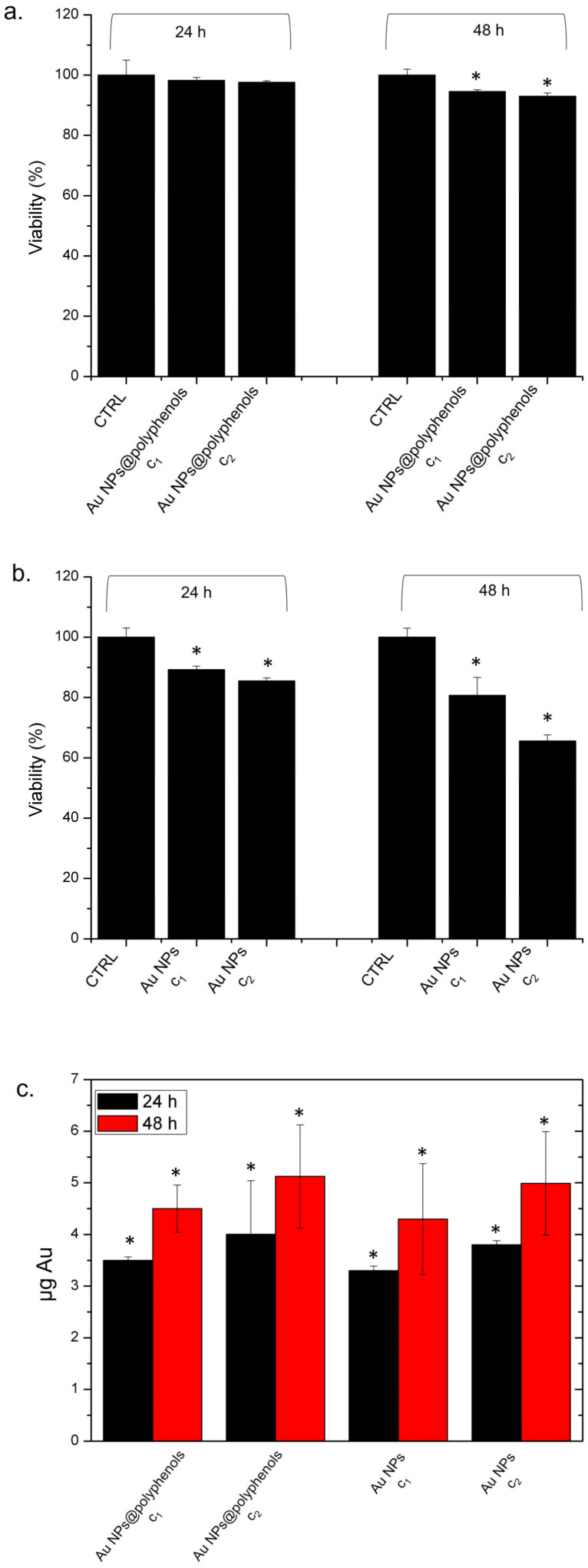
(**a**,**b**) Viability assay (WST-8) of THP-1 cell lines after 24 h and 48 h of exposure to two doses (c1 and c2) of Au NPs@polyphenols (**a**) and conventional Au NPs (**b**). The viability of NP-treated cells was normalized to non-treated control cells. As a positive control (P), the cells were incubated with 5% DMSO (data not shown). Data reported as the mean ± SD from three independent experiments are considered statistically significant, compared with the control (*n* = 8) for *p* value < 0.05 (<0.05 *). (**c**) The accumulation in THP-1 cell lines exposed to c1 and c2 of Au NPs@polyphenols (**a**) and conventional Au NPs for 24 h and 48 h. The cells were then harvested, the live cells were counted, and the Au content was measured in 360,000 cells (μg Au). The control was represented by untreated cells (values = 0, data not shown). The data are reported as the mean ± SD from three independent experiments, and statistically significantly exposed cells vs. control cells have a *p* value < 0.05 (<0.05 *).

The Au NPs@polyphenols were much more tolerated, compared to the standard AuNPs (Figure 2), as we did not observe any decrease in viability after 24 h and only a 7% decrease after 48 h of incubation (Figure 2a). On the other hand, the conventional Au NPs induced severe toxicity, as we observed a decrease of 30% and of 42% of viability after 24 h and 48 h of incubation, respectively (Figure 2b). 

To understand if the difference in viability could be related to different uptake levels, we quantify the NP engulfment by ICP analyses over lysed cells. We did not detect a significant difference between the two types of NPs in terms of internalization (Figure 2c), which remained almost comparable. After 48 h of incubation with Au NPs@polyphenols and conventional Au NPs, we quantified (5.3 ± 1.5) and (5.2 ± 1.8) ug, respectively. The uptake mechanism could be a combination of phago- and endocytosis [68]. 

NF-κB nuclear translocation was used to evaluate the potential pro- or anti-inflammatory effects of Au NPs@polyphenols and conventional Au NPs in M0-macrophages (by immunofluorescence and confocal imaging) [69,70]. 

**Figure 3:**
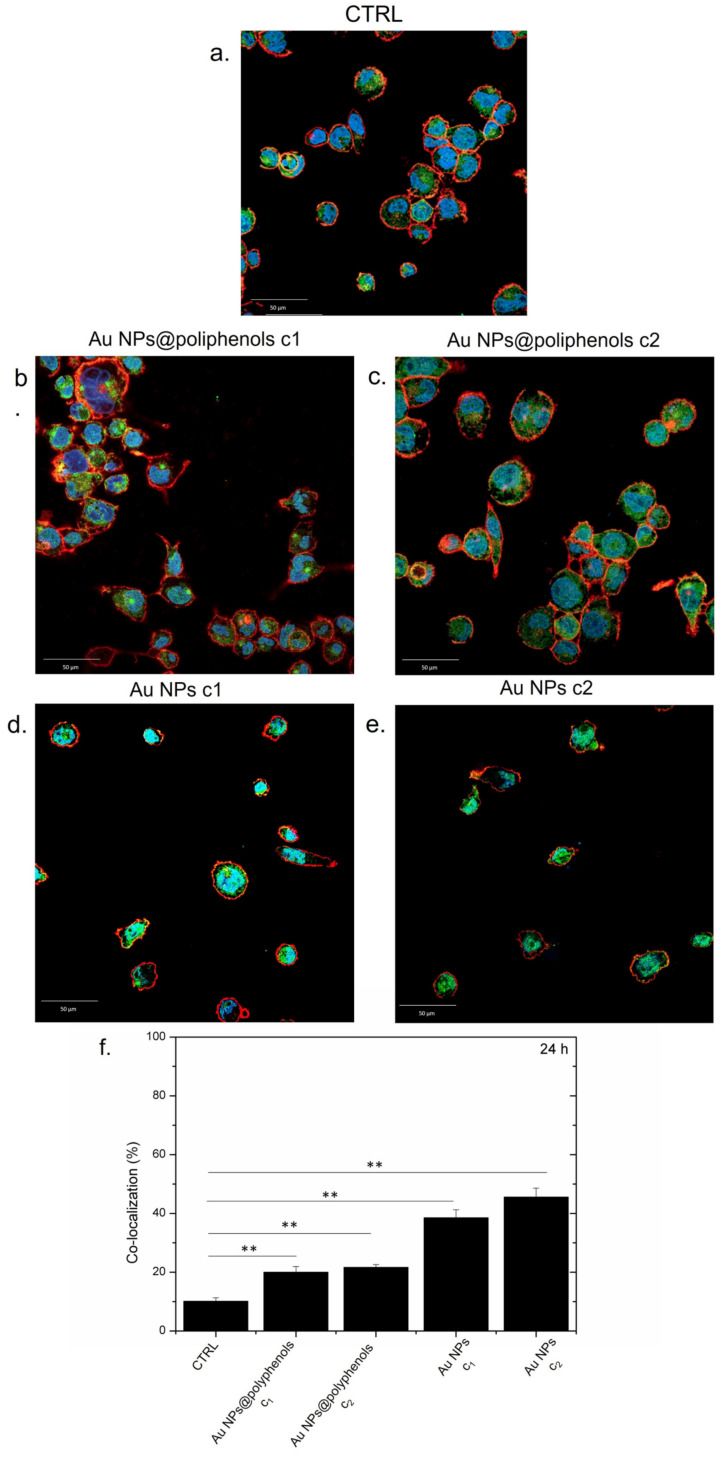
Representative confocal images of untreated macrophages (M0) (**a**) exposed to the c1 and c2 of Au NPs@polyphenols (**b**,**c**) and to the c1 and c2 of conventional Au NPs (**d**,**e**) for 24 h. The cells were fixed and then labeled. The nuclei were stained with Hoechst (blue), Actin cytoskeleton with CellMask™ Deep Red (red), and NF-κB with NF-κB p65 Antibody (F-6) FITC (green intensity signal). (**f**) Co-localization analysis of the merged fluorescence signals due to the NF-κB translocation from the cytoplasm to the nucleus (merged blu/green fluorescence intensity signal). The data are expressed as the mean SD (5 images for *n* = 2) and they were considered statistically significant for ** *p* < 0.01.

**Figure 4:**
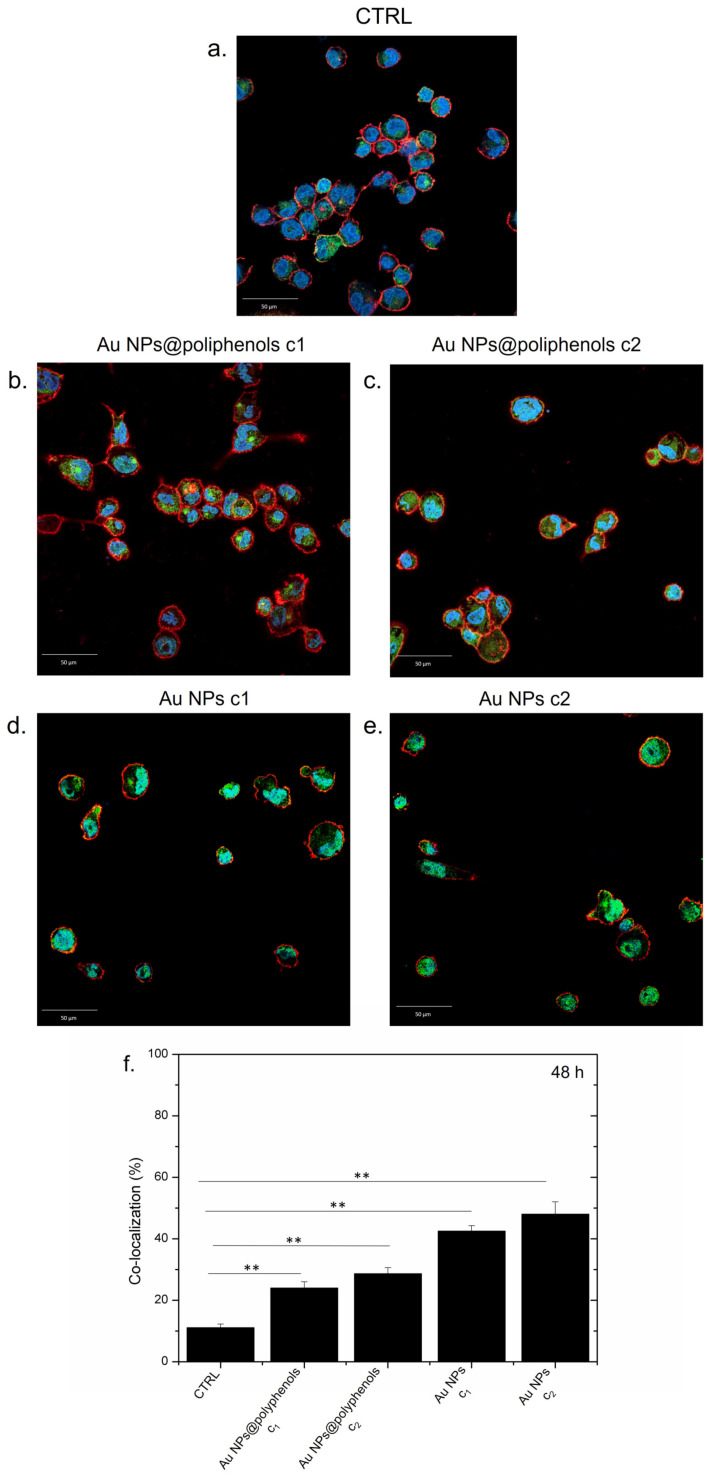
Representative confocal images of untreated macrophages (M0) (**a**) exposed to the c1 and c2 of Au NPs@polyphenols (**b**,**c**) and to the c1 and c2 of Au NPs (**d**,**e**) for 48 h. The cells were fixed and then labeled. The nuclei were stained with Hoechst (blue), Actin cytoskeleton with CellMask™ (red), and NF-κB with NF-κB p65 Antibody (F-6) FITC (green intensity signal). (**f**) Co-localization analysis of the merged fluorescence signals due to the NF- κB translocation from the cytoplasm to the nucleus (merged blu/green fluorescence intensity signal). The data are expressed as the mean SD (5 images for *n* = 2) and they were considered statistically significant for a ** *p* < 0.01.

NF-κB was mostly located in macrophage cytosol in the control (untreated) cells (Figure 3a), indicating no sign of inflammation, as expected. The treatment with Au NPs@polyphenols for 24 h induced only a mild nuclear translocation of NF-κB, compared to the control cells, indicating that the macrophages did not undergo inflammation (Figure 3b,c). The conventional Au NPs, conversely, triggered the transfer of NF-κB to the nucleus in a dose-dependent manner (Figure 3d,e). Quantitative imaging analyses revealed an increase in the co-localization percentage (by Pearson coefficient), which was more relevant for cells exposed to conventional Au NPs (Figure 3f). In particular, Au NPs@polyphenols induced 19% and 23% of nuclear co-localization for the c1 (1 μM) and c2 (3 μM) concentration, respectively. Exposure to conventional Au NPs triggered a greater NF-κB translocation of 38% and 45% using the c1 and c2 concentration, respectively. In particular, the green signal (NF-κB) was mainly placed in the cell’s cytoplasm upon incubation with Au NPs@polyphenols for 24 h and 48 h (Figure 4). Conversely, the green signal co-localized more with DAPI (nuclear staining) after incubation with conventional AuNPs (Figure 4d,e), with a percentage of nuclear NF-κB translocation of about 50% using the highest concentration (Figure 4f). In the case of Au NPs@polyphenols, the values were about 20% with respect to the control sample (c.a 10%) using the highest concentration. The low cell density shown in Figure 3d,e and Figure 4d,e was justified by the results obtained in the viability assay, where the exposure to Au NPs was more toxic than the green counterpart.

NF-κB nuclear translocation is known to trigger the expression of several pro-inflammatory cytokines genes, such as IL-6, IL8, and TNF-α [67,71,72]. Cytokines are small proteins (<40 kDa) that influence and control inflammation. The cytokine release activates cells involved in immune response, inducing the release of further cytokines [73]. IL-6 is involved in the stimulation of the acute phase response, immune reaction, and hematopoiesis [74,75]. In response to various stimuli, IL-6 is synthesized by immune cells, fibroblasts, endothelial cells [76], and in local lesions in the first stage of inflammation. From the bloodstream, IL-6 reaches the liver, where several acute phase proteins (fibrinogen, C-reactive protein, haptoglobin, serum amyloid A, and α1-antichymotrypsin) are stimulated [77]. IL-8, a potent neutrophil chemo-attractant, is secreted by different types of cells, including fibroblasts, monocytes, neutrophils, and endothelial cells [78]. In addition, it is produced by tumor cells, acting as a chemotactic agent for lymphocytes and neutrophils and also inducing an increase of intracellular Ca^2+^ required for migration and phagocytosis proliferation [79]. During acute inflammation, macrophages and monocytes also secrete TNF- α, an inflammatory cytokine and a member of the TNF-superfamily consisting of transmembrane proteins with a homologous TNF domain [80]. TNF-α triggers several signaling pathways, inducing apoptosis or necrosis via Fas and Caspases [81].

**Figure 5:**
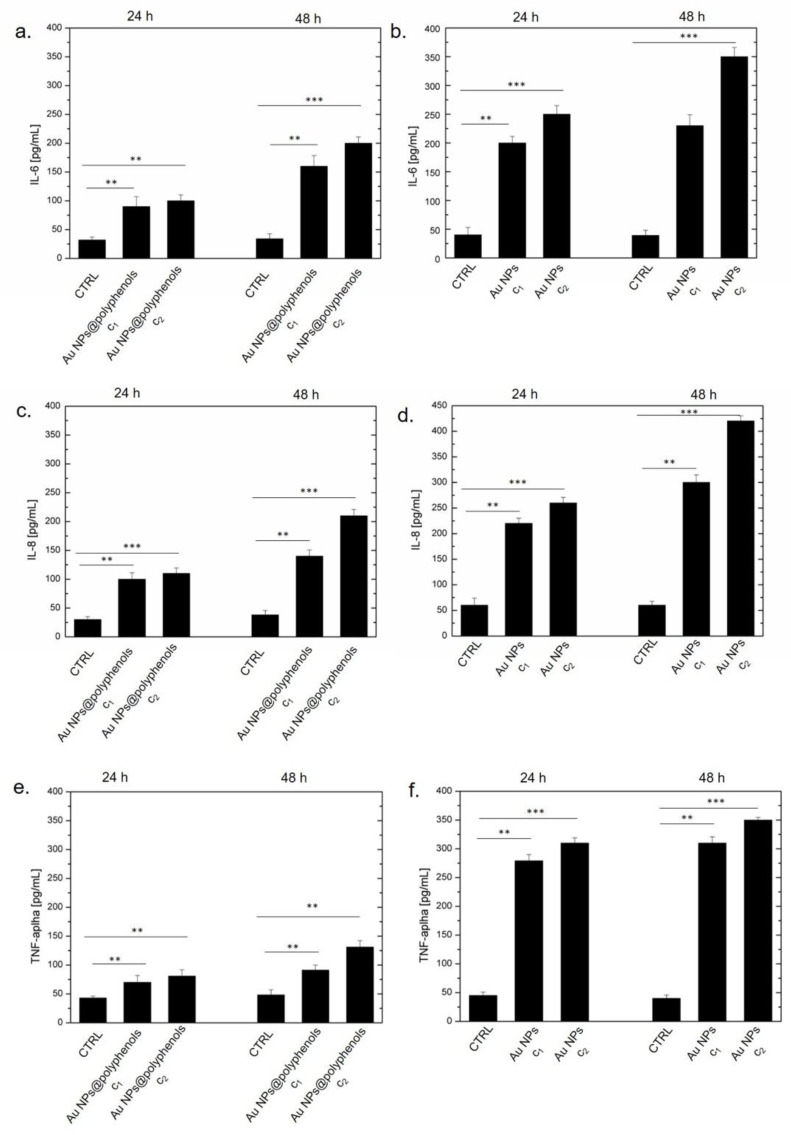
Levels of IL-6 (**a**,**b**), IL-8 (**c**,**d**), and TNF-α (**e**,**f**), expressed as pg/mL. The assessment was conducted by incubating cells with Au NPs@polyphenols and conventional Au NPs at two concentrations (c1 and c2) for 24 h and 48 h. The cytokine levels were detected in supernatants from the control cells and the treated cells by an ELISA assay. The results are expressed as the mean ± standard deviation of three separate experiments. ** *p* < 0.01, *** *p* < 0.001 respect to the control of each time point.

Following the NF-κB activation, we performed ELISA assays over the aforementioned cytokines (in pg/mL) after exposing the macrophages to Au NPs@polyphenols and conventional Au NPs (Figure 5). We detected an IL-6 increase (to 153 pg/mL and 180 pg/mL) when the macrophages were incubated with Au NPs@polyphenols at concentrations of c1 (1 μM) and c2 (3 μM), respectively, for 48 h. Conventional Au NPs triggered, on the other hand, a significant production of IL-6 (400 pg/mL), when cells were treated with 3 μM (c2) for 48 h (Figure 5a,b). Moreover, the cytokine production was time- and dose-dependent. Similar values were found by analyzing the IL-8 production. Additionally, in this case, the Au NPs@polyphenols (c2) induced a secretion of about 200 pg/mL of IL8, while conventional Au NPs induced the production of 440 pg/mL after 48 h (Figure 5c,d).

We also detected an important TNF-α release when the macrophages were incubated with conventional Au NPs for 24 h and 48 h. We quantified about 330 pg/mL for the standard AuNPs and an average value of 130 pg/mL upon exposure to Au NPs@polyphenols (Figure 5e,f).

After investigating whether NPs could trigger inflammation in macrophages as a function of the synthetic route employed, we moved on to exploring their potential application as a heat-based therapy in cancer treatment. During thermal treatment, the polyphenol shell may in fact trigger synergistic polyphenol/Au effects, where the intrinsic anticancer properties of polyphenols can synergistically act together with the high thermal conductivity of Au. In general, thermotherapy is a valuable physical approach to destroying malignant cells when a temperature of about 43 °C is applied for a specific period of time (c.a 45 min) [82]. It is characterized by a low toxicity and the absence of collateral effects, increasing the tumor oxygenation and promoting drug delivery. While this approach has effective features in terms of therapy, the exclusive application of heat is not satisfactory for inducing a complete cancer eradication. This is why heat therapy is mostly used in a combinatorial approach with chemotherapies, which are characterized by very well-established side effects [83]. Nevertheless, in our case, we exploited polyphenols as both an NP-stabilizing agent/shell and chemotherapy replacement.

**Figure 6:**
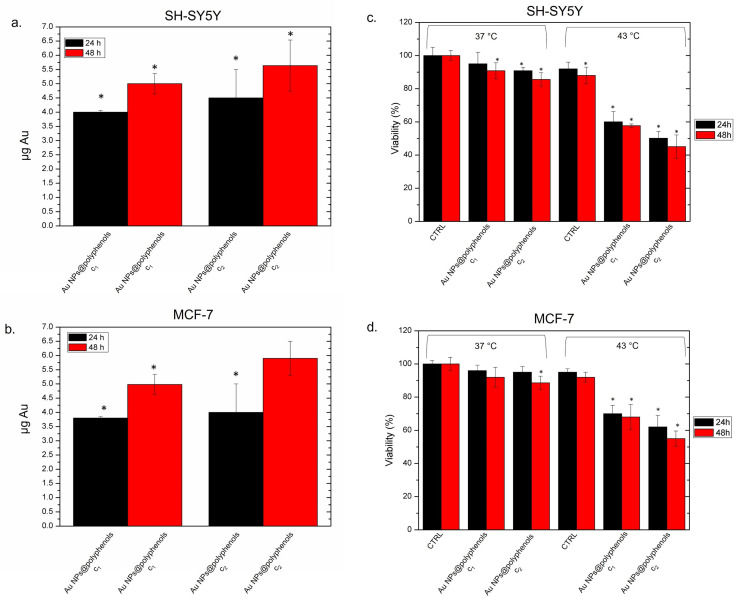
Accumulation in SH-SY5Y (**a**) and MCF-7 (**b**) cell lines exposed to the c1 and c2 of Au NPs@polyphenols for 24 h and 48 h. The cells were then harvested, the live cells were counted, and the Au content was measured in 360,000 cells (μg Au). The data are reported as the mean ± SD from three independent experiments, and statistically significantly exposed cells vs. control cells have a *p* value < 0.05 (<0.05 *). The viability assay (WST-8) of the SH-SY5Y (**c**) and MCF-7 cell lines (**d**) after 24 h and 48 h of exposure to two doses (c1 and c2) of Au NPs@polyphenols at 37 °C and 43 °C, following the procedure described in the section, Materials (ph. 2.10). The viability of NP-treated cells was normalized to non-treated control cells. As a positive control (P), the cells were incubated with 5% DMSO (data not shown). The data reported as the mean ± SD from three independent experiments are considered statistically significant, compared with the control (*n* = 8), with a *p* value < 0.05 (<0.05 *).

We thus assessed the effectiveness of Au NPs@polyphenols against neuroblastoma (SH-SY5Y) and adenocarcinoma (MCF-7) cell lines. We preliminarily quantified the Au NP@polyphenol uptake (Figure 6 a,b) to check if there was a different engulfment rate between the two cell lines. We observed a time- and dose-dependent internalization. In particular, the intracellular Au amount was about 4.5 pg after 24 h and reached 5.5 pg after 48 h in SH-SY5Y cells (Figure 6a). A similar trend was observed for the MCF-7 cells, where exposure to the highest concentration of Au NPs@polyphenols for 24 h led to an intracellular mass of about 4 pg and of 5.8 after 48 h. We concluded that Au NPs@polyphenols shared a similar uptake profile in the two cell lines (Figure 6b). 

Then, the Au NP@polyphenol-based thermal treatment was evaluated over MCF-7 and SH-SY5Y. Commonly, the local thermal treatment is carried out using a temperature ranging from 40 °C to 45 °C for 20–60 min [39]. In our work, we incubated the cells for 45 min at 43 °C with 1 μM and 3 μM (c1 and c2) of Au NPs@polyphenols for 24 h and 48 h to assess a possible synergic effect between the temperature, Au, and polyphenols. In our case, the temperature heating the Au core was characterized by a high thermal conductivity, which, in turn, transmitted the heat to the polyphenols and thus triggered the synergistic anticancer efficiency of the combined therapy.

A reduction in viability was observed in both cell lines after the heat application, with the most severe effects detected in SH-SY5Y. The cells treated only with thermal treatment did not show remarkable effects in terms of cell death (Figure 6c,d). In detail, SH-SY5Y cells exposed to Au NPs@polyphenols (at c1 and c2) displayed 87% of viability at the physiological temperature (37 °C). Upon exposure to thermal treatment (see the materials and methods for details), the viability drastically decreased in a time- and dose-dependent manner. In fact, only 45% of cells were vital after 48 h of exposure at the highest concentration (Figure 6a). The same behavior was observed in MCF-7, where the treatment with NPs did not trigger a considerable loss of cell viability at 37 °C, whereas we observed a 50% decrease in cell viability after 48 h of heat application at the highest NP concentration (Figure 6b). Interesting, untreated cells showed a good tolerance to heat (43 °C) at the two time points tested. These data demonstrated that the temperature treatment alone does not alter cell viability.

**Figure 7:**
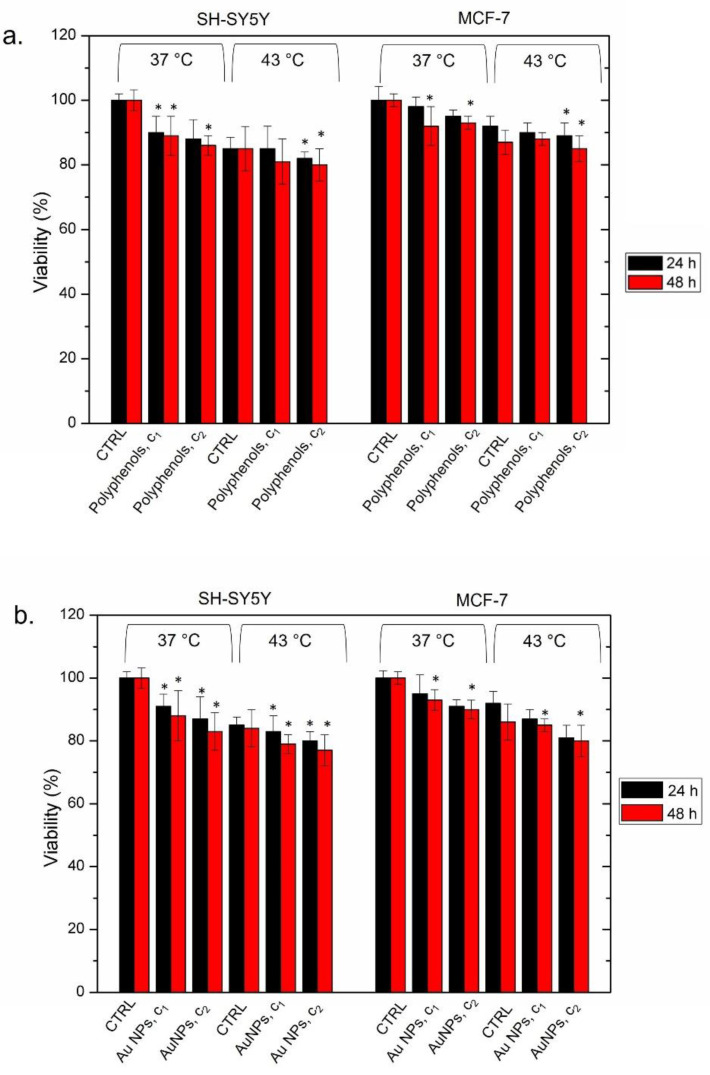
Viability assay (WST-8) of SH-SY5Y and MCF-7 cell lines after 24 h and 48 h of exposure Table 1. and c2) of polyphenols (**a**) and Au NPs (**b**) at 37 °C and 43 °C, following the procedure described in the section, Materials (ph. 2.10). The viability of NP-treated cells was normalized to non-treated control cells. As a positive control (P), the cells were incubated with 5% DMSO (data not shown). The data reported as the mean ± SD from three independent experiments and they are considered statistically significant, compared with the control (*n* = 8), with a *p* value < 0.05 (<0.05 *).

To verify whether cell death was triggered by the combined effect of polyphenols and Au NPs (and not by each single component individually), we separately used them at concentrations of c1 (1 μM) and c2 (3 μM) (Figure 7). 

We did not observe significant changes in terms of viability upon incubating the two cell lines at 37 °C for 24 h and 48 h. The heat treatment, performed in the same way as described above, induced a slight loss of vitality, which was more evident for SH-SY5Y. As a matter of the fact, we found a cell viability of 80% ± 5 after 48 h using the c2 concentration and 43 °C of temperature. The MCF-7 exposed to the same concentration and exposure time exhibited a viability of 85% ± 2 (Figure 7a). The same trend was observed using conventional Au NPs alone. We did not observe a significant cell death in the two cell models, after incubating them at 37 °C and 43 °C for both 24 h and 48 h. The cell viability never fell below 77%, which was reached only by SH-SY5Y heated at 43 °C and incubated with the highest NP concentration (Figure 7b).

These results were indeed not comparable with those observed with Au NPs@polyphenols and heat, where a considerable cell mortality was detected.

**Figure 8:**
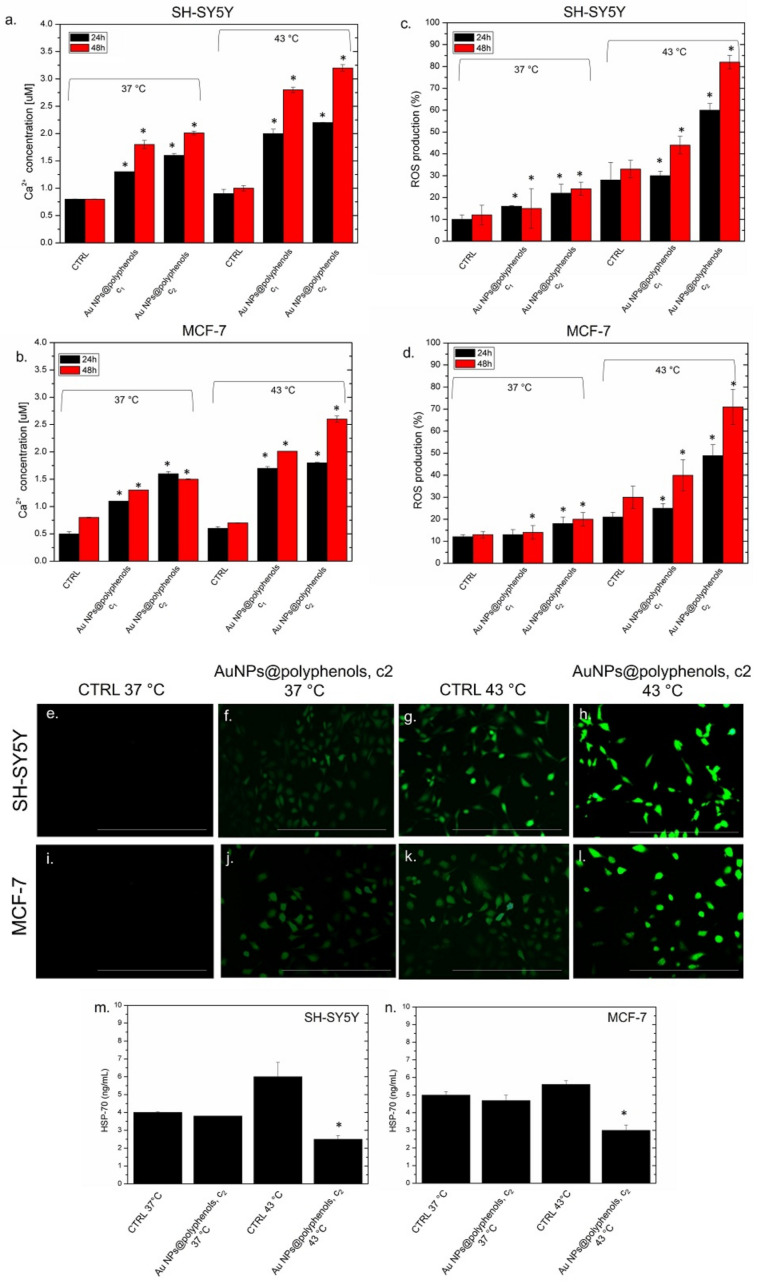
Effect of Au NPs@polyphenols on the Ca^2+^ level in SH-SY5Y (**a**) and MCF-7 (**b**) cells after 24 h and 48 h of exposure to two doses (c1 and c2) of Au NPs@polyphenols at 37 °C and 43 °C, following the procedure described in the section, Materials. The data are reported as the mean ± SD from three independent experiments; * *p* < 0.05, compared with control (*n* = 8). (**c**,**d**) The effect of Au NPs@polyphenols on the ROS level in SH-SY5Y and MCF-7 cells after 24 h and 48 h of exposure to two doses (c1 and c2) of Au NPs@polyphenols at 37 °C and 43 °C, following the procedure described in the section, Materials. The ROS generation of NP-treated cells is expressed relative to non-treated control cells. As a positive control (P), the cells were incubated with 500 μM of H_2_O_2_, showing a ca. 300% DCFH-DA increase (not shown). The data are reported as the mean ± SD from three independent experimens; * *p* < 0.05, compared with the control (*n* = 8). (**e**–**l**) Representative fluorescence images of SH-SY-5Y and MCF-7 cells exposed to the c2 of Au NPs@polyphenols at 37 °C and 43 °C, following the procedure described in the section, Materials. (**m**) ELISA quantitative analyses of HSP70 in SH-SY5Y and MCF-7 cells treated with Au NPs@polyphenols at 37 °C and 43 °C at the c2 concentration, following the procedure described in the section, Materials. Histogram showing the levels of HSP70 expressed in ng/mL in SH-SY5Y (**n**) and MCF-7. The data are presented as the mean ± standard deviation of three independent experiments: * *p* < 0.05.

Then, we quantified the concentration of intracellular calcium (Ca^2+^), which is an ubiquitous intracellular messenger controlling several functions [84]. Generally, an amount of a few Ca^2^ is located in the mitochondrial/nuclear matrix and cytosol, whereas high levels are present in endoplasmic reticulum (ER), in muscle cells, and the sarcoplasmic reticulum [85]. When specific stimuli cause an alteration of this homeostasis, the Ca^2+^ concentration increases in the cytosol. In this condition, apoptosis and necrosis can occur. In our experiments, an alteration in the levels of intracellular Ca^2+^ concentration was analyzed upon exposing SH-SY5Y and MCF-7 to 1 μM (c1) and 3 μM (c2) of Au NPs@polyphenols at 37 °C and 43 °C (Figure 8a,b). We noticed that the incubation of SH-SY5Y to Au NPs@polyphenols at 37 °C (concentration of c1 and c2) induced the production of about (1.8 ± 0.04) and (1.9 ± 0.02) μM, respectively, of Ca^2+^ after 48 h. These values increased when thermal treatment was applied. In particular, the production of intracellular Ca^2+^ reached 3.2 μM with the highest concetration tested (Figure 8a). A similar behaviour was observed in MCF-7 cells, but the effects were weaker. MCF-7 indeed produced (1.5 ± 0.01) μM of Ca^2+^ after 48 h of NP exposure at the c2 concentration, whereas we quantified (2.5 ± 0.08) μM of Ca^2^ after 48 h. Since an excess of intracellular Ca^2+^ can trigger ROS formation, we investigated if oxidative stress was induced by the combined treatment of Au NPs@polyphenols and heat. In particular, we demonstrated that the Au NP@polyphenol treatment, or the heat application, did not individually cause dangerous levels of ROS production in SH-SY5Y and MCF-7 (Figure 8c,d). The heat induction (ctrl 43 °C) led to a 30% increase of ROS, compared to the control (cells wihout NP incubation at 37 °C), in which the percentage of ROS recorded was about 10% using the DCFH-DA assay. On the contrary, the synergic action of Au NPs@polyphenols and heat enhanced the intracellular presence of ROS. In particular, we quantified an ROS production of 80% in SH-SY5Y after 48 h of incubation with NPs at the c2 concentration (Figure 8c). This value was 70% in the MCF-7 cells (Figure 8d). These results were confirmed using fluorescence microscopy (Figure 8e–i), where an increase in green fluorescence can be noted, corresponding to the ROS concentration enhancement of cells treated with NPs and heat at the same time (c2, 48 h). The ROS production was more pronounced in SH-SY5Y than MCF-7 (Figure 8h,l). The cells treated with the c2 of Au NPs@polyphenols at the physiological temperature (37 °C) (Figure 8f,j) showed only a slight increase of fluorescence. The combination of thermal treatment and oxidative stress can induce further responses involving Heat Shock Protein (HSP) production, such as HSP-70 [86]. The role of HSP-70 is crucial in the repair of denatured proteins, as it stops their misfolding and/or undesired aggregation [87]. HSP-70 is overexpressed in most human cancer cells, contribuing to tumor development [88,89,90]. HSP-70 also mediates the resistance to therapies and contributes to the development of an aggressive tumour phenotype [91]. However, a deficiency, or excess, of antioxidants can modulate the activation (or suppression) of HSP-70 synthesis [92]. To examine the effects of Au NPs@polyphenols and heat on HSP-70 production, we heated (or not) the SH-SY5Y and MCF-7 with the c2 concentration of NPs for 48 h. As a control, untreated cells grew at the physiological temperature, and the cells were exposed to heat (43 °C). A basal level of HPSP-70 was observed for the untreated cells at 37 °C, since the tumor cells normally overexpressed this class of proteins [90] (Figure 8m,n). The treatment with Au NPs@polyphenols did not induce evident alterations of the HSP-70 release, compared to the control, in both the cell lines at 37 °C. Conversely, an increase in the HSP-70 production was observed in the cells incubated at 43 °C. The HSP-70 amount was about 6 ng/mL in SH-SY5Y, compared to the control, at 37 °C (c.a 3 ng/mL) (Figure 8m). This value was slightly lower in the MCF-7 cells (5.5 ng/mL), but in this case, the control at 37 °C showed a basal quantity of about 5 ng/mL (Figure 8n). On the other hand, the HSP-70 induction was strongly reduced to 2.3 ng/mL and 3 ng/mL by the heat and NP treatment in SH-SY5Y and MCF-7 (Figure 8m,n). These results are in agreement with previous works, where NPs-polyphenols were used in cancer cells [93,94] and triggered the release of the HSP family, followed by the activation of anti-tumoral pathways [95]. In our work, the synergic effect of Au, polyphenols, and heat enhanced the inactivation of HSP-70, a critical aspect in cancer therapy applications.

**Figure 9:**
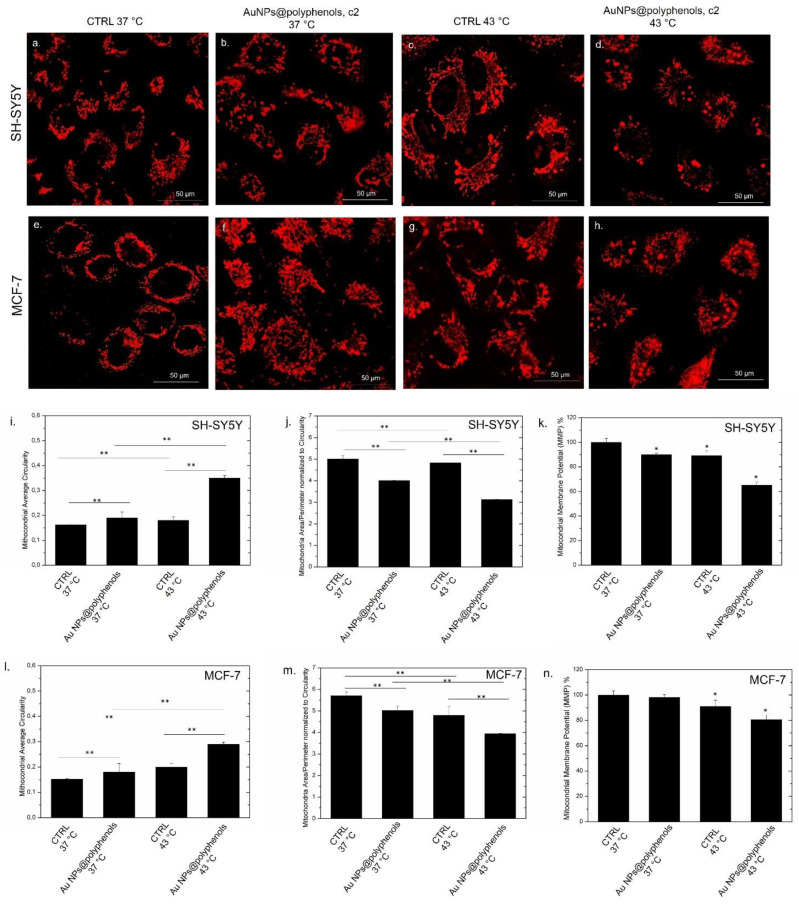
Confocal microscope acquisitions of SH-SY5Y and MCF-7 mitochondria labelled with MitoTracker™ Deep Red^FM^. Untreated cells (**a**,**e**), cells exposed to Au NPs@polyphenols at the concentration of c2 at 37 °C (**b**,**f**), cells treated with 43 °C (**c**,**g**), and cells incubated with Au NPs@polyphenols at the concentration of c2 at 43 °C (**d**,**h**). The mean values and standard deviations of the mitochondrial average circularity of SH-SY5Y (**i**), MCF-7 (**l**), and mitochondria area/perimeter normalized to the circularity of SH-SY5Y (**j**) and MCF-7 (**m**) treated as in (**a**–**e**). The effect of Au NPs@polyphenols on the Mitochondrial Membrane Potential (MMP) level in SH-SY5Y (**k**) and MCF-7 cells (**n**) after 24 h and 48 h of exposure to two doses (c1 and c2) of Au NPs@polyphenols at 37 °C and 43 °C, following the procedure described in the section, Materials. After 48 h, the cells were incubated with JC-1, and the fluorescence of the cells from each well was measured and recorded. The percentage mitochondrial membrane potential of nanoparticle-treated cells was expressed relative to the untreated control cells. As a positive control (P), the cells were incubated with 100 μM of valinomycin. The data are reported as the mean ± SD from three independent experiments; * *p* < 0.05, ** *p* < 0.001 compared with control (*n* = 8). The data are presented as the mean ± standard deviation of three independent experiments:.

The mitocondrial damage is also stricly linked with the oxidative stress and calcium release. In particular, the intracellular Ca^2+^ overload induces the swelling and collapse of the mitochondrial membrane potential [96]. In order to test if NPs triggered such a condition as well, we performed confocal analyses on labeled SH-SY5Y and MCF-7 mithocondria co-treated with Au NPs@polyphenols and heat (Figure 9a–h). The results showed significant morphological alterations with respect to untreated cells at 37 °C (Figure 9a,e), which displayed physiological mitochondrial morphologies instead. The shape became shorter and more circular when heat and Au NPs@polyphenols (Figure 9d,h) were applied, indicating a loss of structural integrity and swelling with respect to cells treated with either NPs (Figure 9b,f) or heat at 43 °C (Figure 9c,g). The “Mito Morphology” macro text plug-in of the ImageJ software [97] provided an estimation of the circularity (index of elongation) of SH-SY5Y and MCF-7 mitochondria (Figure 9i,l), assuming that values near 1 correspond to an ideal perfect cicle. Untreated cells displayed average circularity values of c.a. (0.15 ± 0.003) for SH-SY5Y mitochondria and (0.14 ± 0.002) for those of MCF-7, showing an elongated shape. The circularity was similar to the control at 37 °C, after treatment (or not) with Au NPs@polyphenols. On the other side, the synergistic effect caused by Au NPs@polyphenols and heat induced an ehancement of the mitocondrial circularity in both cell lines, which had a more evident spherical shape. This alteration was greater in SH-SY5Y (circularity value equal to (0.35 ± 0.01) (Figure 9i), compared to MCF-7 (circularity value equal to (0.28 ± 0.03) (Figure 9l). The mitochondria morphology change induced by the combination of AuNPs@polyphenols and heat was also reported as the mitochondrial average area/perimeter ratio (Figure 9j,m). This was normalized to the circularity to account for swollen mitochondria that attain a large area. We quantify values of c.a. (5.0 ± 0.2) and (5.6 ± 0.2) for untreated SH-SY57 and MCF-7 at 37 °C. The parameter slightly decreased down to (4 ± 0.5) and (5 ± 0.08) after exposure to Au NPs@polyphenols at 37 °C and switched to (4.8 ± 0.04) and (4.6 ± 0.005) after heat application for SH-SY5Y and MCF-7, respectively. The values strongly decreased to (3 ± 0.2) for SH-SY5Y (Figure 9j) and (3.8 ± 0.5) for MCF-7 (Figure 9m) when the synergic effect was triggered by the coupled effect of Au NPs@polyphenols and heat. Our data demonstrated that the mitochondria underwent a morphology transformation to a circular compact shape, followed by fragmentation due to intracellular oxidative stress. We also investigated the possible alteration of mitochondrial membrane potential (MMP) by analyzing the J-aggregate—JC1 dye. As expected, the alteration of the mitochondria morphology (due to the increase of ROS and Ca^2+^ levels) also had adverse effects on the mitochondrial potential homeostasis in the treated cells. In particular, the MMP percentage remained approximatively similar to the control (untreated cells at 37 °C) in cells alternatively exposed to NPs at the c2 concentration or heat for 48 h. Conversely, the synergistic treatment exerted by NPs and heat induced a decrease to c.a 63% and 78% for SH-SY5Y and MCF-7, respectively.

Perturbations of the MMP, assessed by the JC-1 assay, were found to be particularly evident when the Au NPs@polyphenols and heat were used simultaneously. In good agreement with previous experiments, it was clear that there was a connection between Ca^2+^ overload, oxidative stress, and mitochondria collapse. 

**Figure 10:**
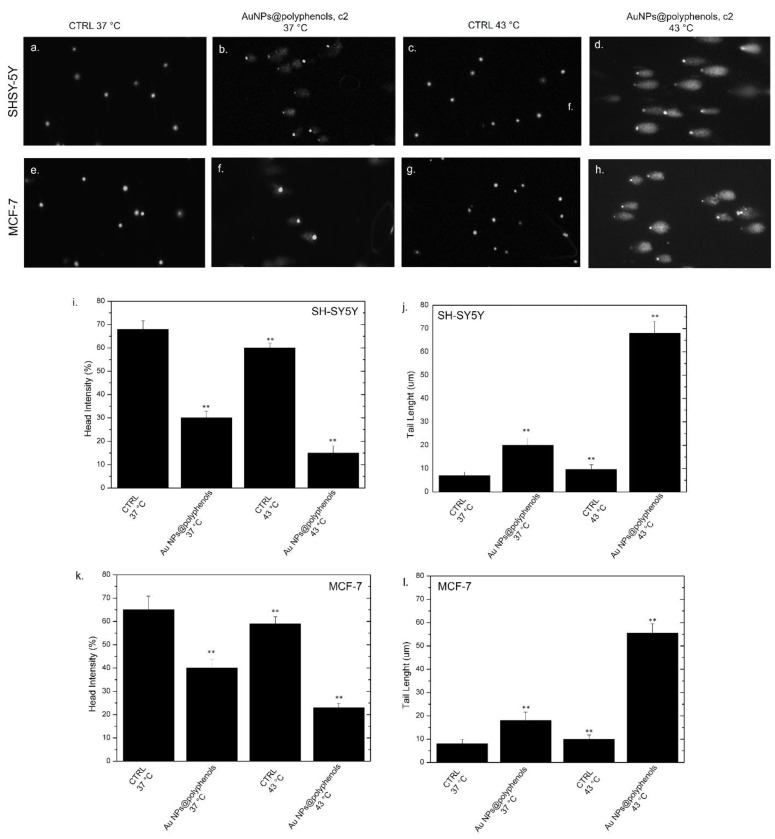
Representative images of DNA damage in SH-SY5Y (**a**–**d**) and MCF-7 (**e**–**h**). Untreated cells (**a**,**e**) exposed to AuNPs@polyphenols at the concentration of c2 at 37 °C (**b**,**f**), treated with 43 °C (**c**,**g**), and incubated with Au NPs@polyphenols at the concentration of c2 at 43 °C for 48 h. The DNA damage was evaluated by the head intensity (%) (**i**,**k**) and tail length (μm) (**j**,**l**). The values shown are the means from 100 randomly selected comet images of each sample. As a positive control, the cells were incubated with 500 μM of H_2_O_2_ (data not shown). The data are reported as the mean ± SD from three independent experiments; ** *p* < 0.001 compared with control (*n* = 3).

We then finally explored whether the heat shock could trigger DNA and chromosomal damage, with the potential inhibition of DNA repair in cancer cells. We thus investigated the effect of heat and heat/Au NPs@polyphenols on the DNA of SH-SY5Y and MCF-7 by the comet assay, both in terms of the tail length and DNA percentage in the head (Figure 10a–h). Our results indicated that exposure to Au NPs@polyphenols at 37 °C induced a decrease of the head intensity in SH-SY5Y cells (c.a. 30%), with respect to the untreated cells, at 37 °C (c.a 68%) (Figure 10i). These data were opposite to those for the tail length, showing a slight increase of this parameter in cells treated with Au NPs (20 ± 0.03) μm, with respect to the untreated cells, at 37 °C, where the comet morphology was absent (8 ± 0.03) μm (Figure 10j). Upon heating to 43 °C, both the head intensity and tail length values were similar to the control cells at 37 °C. On the other hand, the application of heat in cells previously incubated with Au NPs@polyphenols displayed a strong decrease in the head intensity (c.a 18%) corresponding to a robust tail length growth of about (70 ± 0.04) um (Figure 10i,j).

A similar behavior was noted in the MCF-7 cells, but with slightly stronger effects. For example, the application of heat and NP incubation induced a reduction in the head intensity percentage (c.a. 25%) (Figure 10k) and an increase in the tail length of about 55% (Figure 10l), indicating DNA damage. Additionally, in this case, the thermal treatment did not stimulate evident damage in the control cells both at 37 °C and 43 °C, whereas a few adverse effects were measured after the uptake of Au NPs@polyphenols at 37 °C. 

**Figure 11:**
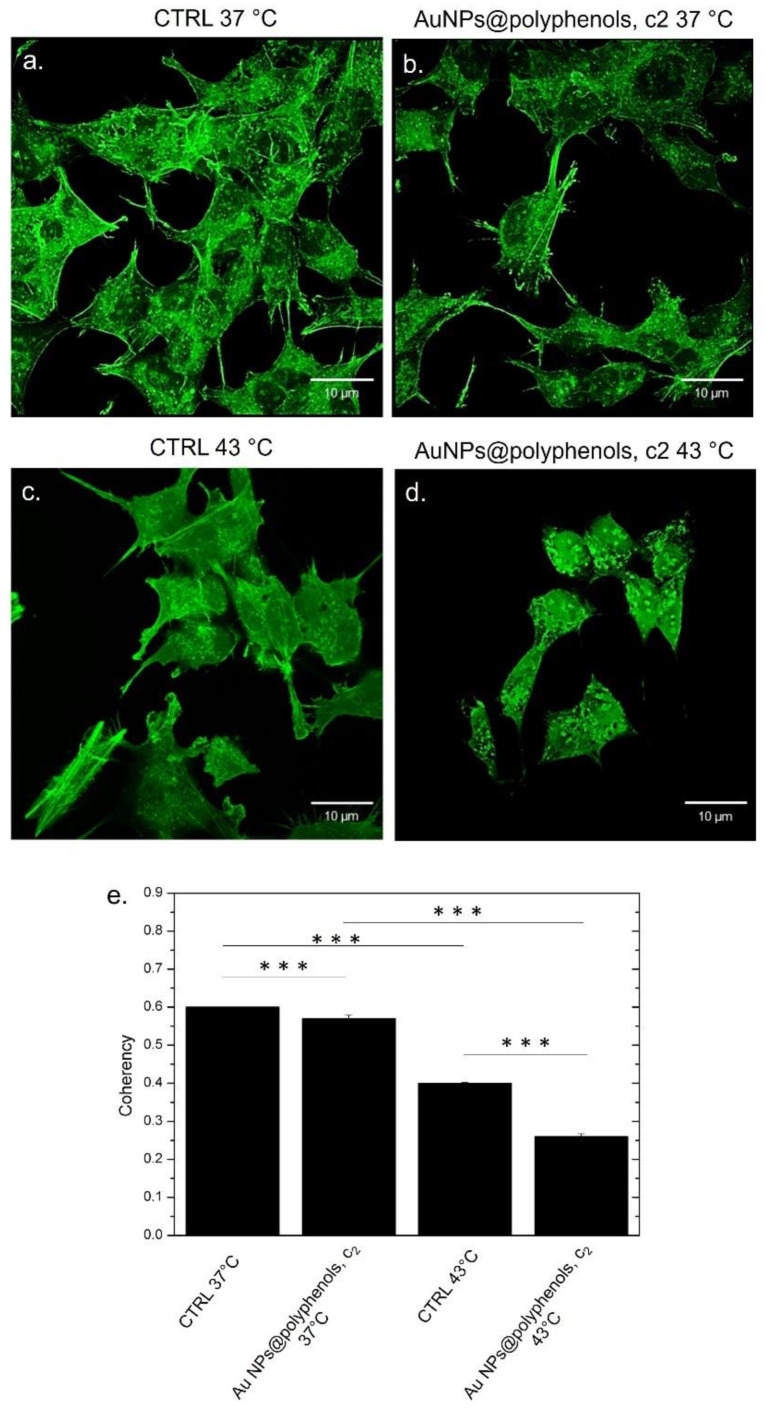
Effect of Au NPs@polyphenols on the actin network in SH-SY5Y exposed to the c2 of Au NPs@polyphenols at 37 °C and 43 °C for 48 h, following the procedure described in the section, Materials (ph. 2.10). (**a**) untreated cells at 37 °C; (**b**) cells incubated with Au NPs@poyphenols at c2 and 37 °C; (**c**) cells treated with 43 °C; (**d**) cells treated with heat and Au NPs@polyphenols at 43 °C. The cells were fixed and then stained with Phalloidin–FITC. 2D images of cortical actin were acquired using a Zeiss LSM700 (Zeiss) confocal microscope, equipped with an Axio Observer Z1 (Zeiss) inverted microscope, using a ×100, 1.46 numerical aperture oil immersion lens. All data were processed using the ZEN software. (**e**) The coherency values calculated on the confocal acquisitions (**a**–**d**) were expressed as the mean value and relative SD using ImageJ (calculation on 15 cells). The mean values and their standard deviations are reported in the histograms. Data were statistically significant for *** *p* < 0.0001.

**Figure 12:**
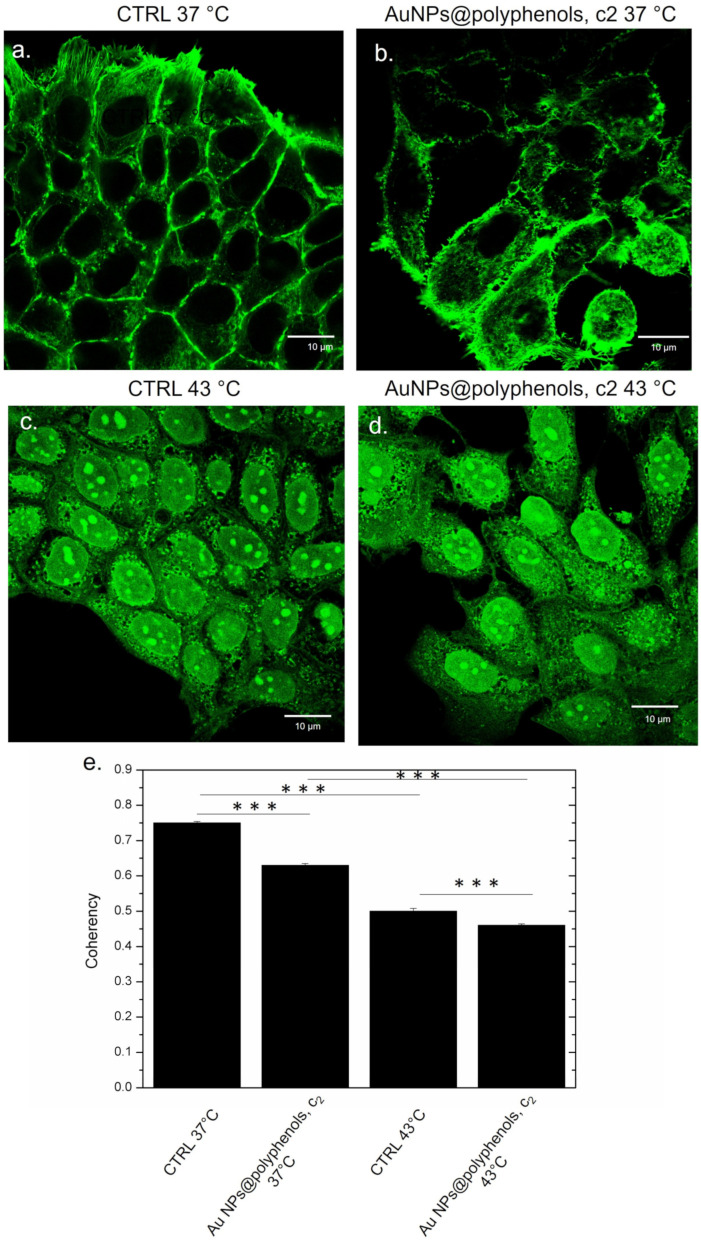
Effect of Au NPs@polyphenols on the actin network in MCF-7 exposed to the c2 of Au NPs@polyphenols at 37 °C and 43 °C for 48 h, following the procedure described in the section, Materials (ph.2.8). (**a**) Untreated cells at 37 °C; (**b**) cells incubated with Au NPs@poyphenols at c2 and 37 °C; (**c**) cells treated with 43 °C, (**d**) cells treated with heat and Au NPs@polyphneols at 43 °C. The cells were fixed and then stained with Phalloidin–FITC. 2D images of cortical actin were acquired by a Zeiss LSM700 (Zeiss) confocal microscope, equipped with an Axio Observer Z1 (Zeiss) inverted microscope, using a ×100, 1.46 numerical aperture oil immersion lens. All data were processed using the ZEN software. (**e**) The coherency values calculated on the confocal acquisitions (**a**–**d**) were expressed as the mean value and relative SD using ImageJ (calculation on 15 cells). The mean values and their standard deviations are reported in the histograms. Data were statistically significant for *** *p* < 0.0001.

The impairment of DNA and mitochondrial functions can have several effects on the cytoskeleton, which is characterized by a dense polymer meshwork that gives shape to the cell, thus contributing to the integrity of the cell membrane [98]. We therefore evaluated the possible alteration of the cortical actin on cells treated as described in previous experiments to strengthen the hypothesis of our system as a powerful antitumor tool. We then stained the actin with phalloidin-FITC (Figure 11 and Figure 12) and performed confocal analyses. A completely different morphology was evident between the treated and control cells. The control SH-SY5Y displayed the typical neuroblast-like morphology at 37 °C after 48 h, with extending neurites in their surrounding area. In addition, the actin fibers appeared to be well organized, and the cells were interconnected with each other (Figure 11a). The cells exposed to the Au NPs@polyphenols at a concentration of 3 μM (c2) did not show dissimilarity, compared to the control (Figure 11b). Differences were observed when a temperature of 43 °C was applied. The heat alone triggered actin random reshuffling (Figure 11c), whereas the synergic effects of Au NPs@polyphenols and 43 °C induced remarkable damage to the actin fibers, which appeared fairly curled. In addition, the cells lost their typical morphology (Figure 11d). The altered organization of the actin network upon NP and heat exposure was quantitatively analyzed by the coherency parameter using ImageJ. The coherency parameter gives information on the degree of fiber orientation, compared to the surroundings. The control samples had a value of (0.62 ± 0.03) after 48 h of treatment at 37 °C (Figure 11e), which changed to (0.58 ± 0.04) for SH-SY5Y exposed to Au NPs@polyphenols at the concentration of c2. The values were very similar, confirming the morphological characteristics observed in the confocal images. When heat was applied, the coherency decreased to (0.43 ± 0.05) and to (0.26 ± 0.03) after the Au NP@polyphenol/heat treatment, which corresponds to a disordered actin configuration. 

Similar results were obtained for MCF-7 (Figure 12). The control cells (at 37 °C) displayed the typical epithelial shape, showing a well-defined actin architecture with organized filaments (Figure 12a). A similar morphology was observed in samples treated with Au NPs@polyphenols, even if a slightly more cytoskeleton disorganization was noted in some area with respect to the control (Figure 12b). On the other hand, the heat induced an alteration of the cytoskeleton (Figure 12c), which was more evident in cells simultaneously treated by 43 °C and NPs, showing a loss of adherent adhesions and a less orderly actin network (Figure 12d). Additionally, in this case, the coherency value measurements corroborated the confocal investigations. The enhancement of actin changes was stronger in cells treated both with heat and NPs, observing a value of (0.44 ± 0.05), with respect to the control at 37 °C (0.75 ± 0.04) and cells treated with 43 °C (0.52 ± 0.07) (Figure 11e).

## 4. Conclusions

NPs synthesized using a green chemistry approach were proven to be safer for cells, as they did not induce a significant inflammation response in activated macrophages. On the other hand, they showed crucial anticancer features. The use of Au NPs@polyphenols in cancer nanomedicine can indeed have several advantages in terms of the production procedures. Firstly, NPs were synthesized by a green route, which means no toxic and/or harmful wastes were produced. Secondly, the typical post-production purification processes required to remove undesired compounds adsorbed on the surface of NPs can be avoided. In this proof-of-concept study, we in fact achieved spherical Au NPs with a small size, surrounded by a visible shell of polyphenols, using a one-step method. We finally demonstrated that polyphenols, in combination with Au NPs and thermal application (43 °C), can have a toxic behavior towards two types of ca ncer cells due to a synergistic action that does not occur if we use either polyphenols or AuNPs individually or heat alone. The high thermal conductivity of Au indeed allows for a quick environmental heat up, transmitting this heat to the polyphenols, which in turn act as anticancer agents. The future challenge will be to test these nanomaterials in animal models.

## Figures and Tables

**Table 1:** Total polyphenol measurements in *Laurus nobilis* leaf extract and in Au NP@polyphenol water solution (5 μM of Au).

Samples	Leaves Extract	AuNPs@polyphenols Solution
Total polyphenols (mg/L)	27 ± 3.7	15 ± 2.4

**Table 2:** Characterization of Au NPs@polyphenols and Au NPs (achieved by conventional chemical route) in water, DMEM, and RPMI by DLS and ζ-potential (mV) measurements.

**Samples in Water**	**Size (nm)**	**ζ-potential (mV)**
Au NPs@polyphenols	28 ± 5	−48 ± 3
Au NPs	20 ± 2	−40 ± 4
**Samples in DMEM**	**Size (nm)**	**ζ-potential (mV)**
Au NPs@polyphenols	32 ± 5	−50 ± 2
Au NPs	32 ± 7	−45 ± 6
**Samples in RPMI**	**Size (nm)**	**ζ-potential (mV)**
Au NPs@polyphenols	30 ± 2	−53 ± 3
Au NPs	33 ± 4	−48 ± 5

## Data Availability

The data presented in this study are available in this article.
